# Origin, phenotype and autoimmune potential of T cells in human immune system mice receiving neonatal human thymus tissue

**DOI:** 10.3389/fimmu.2023.1159341

**Published:** 2023-05-12

**Authors:** Tara Talaie, Hui Wang, Wan-I Kuo, Nichole Danzl, Mert R. Gulsen, Amber N. Wolabaugh, Xiaolan Ding, Megan Sykes, Hao Wei Li

**Affiliations:** ^1^ Columbia Center for Translational Immunology, Department of Medicine, Columbia University, New York, NY, United States; ^2^ Department of Surgery and Department of Microbiology & Immunology, Columbia University, New York, NY, United States

**Keywords:** human immune system mouse model, neonatal thymus, cord blood CD34 + cells, thymopoiesis, autoimmune disease

## Abstract

Robust human immune system (HIS) mice are created using human fetal thymus tissue and hematopoietic stem cells (HSCs). A HIS mouse model using neonatal human thymus tissue and umbilical cord blood (CB) HSCs (NeoHu) was recently described. We improved the model by removing the native murine thymus, which can also generate human T cells, and demonstrated definitively the capacity of human T cells to develop in a grafted neonatal human thymus. Human T cells derived from the neonatal thymus tissue appeared in peripheral blood early post-transplantation and CB-derived T cells appeared later. Naïve T cells were demonstrated in peripheral blood but effector memory and T peripheral helper phenotypes predominated later, in association with development of autoimmunity in some animals. Treatment of thymus grafts with 2-deoxyglucose (2-DG) increased the proportion of stem cells derived from injected HSCs, delayed onset of autoimmune disease, reduced early T cell reconstitution, and reduced effector/memory T cell conversion. Younger neonatal human thymus tissue was associated with improved T cell reconstitution. While the NeoHu model bypasses the need for fetal tissue, it has yet to demonstrate equivalent reconstitution to fetal tissue, though 2-DG can improve results by removing native thymocytes prior to transplantation.

## Introduction

The availability of highly immunodeficient, NOD-scid-common gamma chain deficient (NSG) mice, that lack murine T, B and NK cells, has greatly enhanced the ability to generate human immune system (HIS) mice. One of the critical components for generating HIS mice with optimal immune function is the availability of fetal human thymus tissue. Fetal human thymus tissue supports robust human thymopoiesis from injected fetal (1) or adult (2) CD34^+^ cells. These maintain a steady supply of T cell progenitors to the thymus and generate B cells, dendritic cells (DCs), and monocytes in the bone marrow. The latter cell types serve as antigen-presenting cells (APCs) for T cells in the periphery (1, 3, 4).

T cells developing *de novo* in the fetal human thymus graft are tolerant of the murine host, presumably due to deletion by murine APCs that are detectable in these grafts (5). While the native murine thymus is capable of generating human T cells at a low level, the abnormal structure of the murine thymus results in a failure of normal negative selection (6). This, combined with slow peripheral T cell reconstitution and consequently high levels of lymphopenia-induced proliferation (LIP), results in a severe autoimmune syndrome that can be prevented by native mouse thymectomy (6). In contrast, the implantation of human fetal thymus tissue in HIS mice receiving CD34^+^ hematopoietic stem/progenitor cells (HSPCs) results in a human thymus with normal structure (6). This human fetal thymus achieves relatively rapid reconstitution of naïve human T cells in the periphery (7), with markedly reduced LIP and markedly delayed autoimmunity compared to that observed for T cells developing in the native NSG mouse thymus (6).

In view of problems with the availability and use of human fetal tissue, it is desirable to identify another source of thymic tissue that could function similarly to that from human fetuses. While considerable interest was generated by a recent report using neonatal human thymus grafts, these grafts did not grow and very few naïve T cells (CD45RA^+^CCR7^+^) were detected in the periphery, possibly reflecting extensive peripheral LIP of a small number of T cells originating from the native mouse thymus and/or the grafted thymic tissue, which contained too few thymocytes to count at the time of sacrifice (8). In contrast, fetal thymus grafts grow markedly to become as large as or larger than the kidney itself and can generate up to hundreds of millions of human thymocytes (1–3, 9–13). Thus far, no published data have shown that post-natal human thymus could achieve a similar level of *de novo* thymopoiesis from injected HSPCs.

In this paper we have attempted to reproduce and improve upon the HIS mouse model reported by Brown et al. (8) and to further characterize it. We focus on several important issues that were not fully addressed in Brown’s report. These include the fact that Brown et al. did not thymectomize the murine recipients, whose native thymi can support human T cell development, precluding any definitive demonstration that human T cells developed in the grafted neonatal human thymus. Furthermore, we now dissect the origin of human T cells from the neonatal thymus tissue vs the injected CB CD34^+^ cells and address the development of autoimmune disease in this model as well as the ability of neonatal thymic tissues of different ages to support human T cell reconstitution. To this end, we evaluate and characterize human immune cell reconstitution in a HIS mouse model constructed with neonatal human thymic tissue and umbilical cord blood or fetal liver-derived CD34^+^ HSPCs in thymectomized recipients (NeoHu Model). For the first time, we show definitively that human T cells developing in neonatal thymic grafts rather than the mouse thymus can populate the peripheral immune systems of NSG mice, However, we also show that many of these T cells originate in the grafted neonatal thymus itself rather than being derived from the injected CB CD34^+^ cells. We demonstrated the propensity of these T cells to induce autoimmune disease in the recipients in association with effector/memory phenotypic conversion. Furthermore, we demonstrate the ability of thymocyte depletion with 2-deoxy-D-glucose (2-DG) prior to tissue implantation to delay autoimmune disease development and reduce human T cell reconstitution by cells carried within the neonatal thymus tissue. Finally, we evaluate the effect of neonatal thymus tissue age on T cell reconstitution and phenotype.

## Materials and methods

### Animals and tissues

NSG (NOD.Cg-Prkdc^scid^ Il2rg^tm1Wjl^) mice purchased from Jackson Laboratory (Bar Harbor, ME) were housed in a specific pathogen-free environment. Neonatal human thymus tissue was obtained from discarded pediatric thymic tissue from cardiac surgeries performed at New York Presbyterian Hospital, New York, NY. Human umbilical cord blood (CB) was obtained from the New York Blood Center (New York, NY) or Upstate Cord Blood Bank (Syracuse, NY). Discarded human fetal liver tissues (gestational age 17 to 20 weeks) were obtained from Advanced Biosciences Resource. Studies were conducted with approval by the Institutional Review Board and Animal Care and Use Committees at Columbia University. Further methodological details are available in the **Supplementary Material**.

### Generation of HIS mice

6-8-week-old female NSG mice were thymectomized as we have previously described (7). Two weeks later, these mice received sublethal total body irradiation (1 Gy) by an X-Ray irradiator (RS-2000, Rad Source Technologies, Inc., Suwanee, GA) followed by surgical implantation of a cryopreserved and thawed 2-4mm^3^ human neonatal thymic fragment either bilaterally, for a total of two thymic tissue pieces per mouse or under one kidney capsule. Prior to surgical implantation the thawed thymic tissue was agitated repeatedly with a pipette to dislodge residual thymocytes and, in some experiments (as detailed in **Table 1**), was incubated with 2-DG (Carbosynth, Newbury, UK) for 16-18 hours prior to transplantation for further removal of pre-existing thymocytes. For Experiments 1, 2, and 4, frozen CB-derived magnetically isolated CD34^+^ cells (1-2×10^5^/mouse) were injected intravenously. A total of 3 different CB’s were used in Experiment 1, and 2 different CB’s were used in Experiment 2. In Experiment 3, CD34^+^ cells were isolated from human fetal liver cells (FLCs) in the same manner. Anti-CD2 monoclonal antibody (400µg/mouse) was injected intraperitoneally once a week for 2 weeks (Days 0, 7 and 14) post-transplantation. Further methodological details are available in the **Supplementary Material**.

**Table 1:** Experiments.

Experiment	N	2-DG treatment	Source of CD34^+^ HSCs	Age of neonatal human thymi
1	19	No	CB	2 days old
2	7	Yes	CB	2 days old
3	15 (n=5 for each age)	Yes	FLCs	2 weeks, 2 months, and 4 months old
4	10	Yes	CB	3 days old

The specific details of the 4 cohorts of HIS mice generated using neonatal human thymus, either treated or not treated with 2-DG, plus CD34^+^ HSPCs derived from either CB cells or FLCs, are detailed in **Table 1**.

### Flow cytometry

Every 4 weeks following transplantation, white blood cells (WBCs) from the HIS mice were obtained from the tail vein and flow cytometry was performed to assess levels of human immune cell reconstitution. Percentages and absolute counts of each population were analyzed as previously described (7). Absolute numbers of each population were calculated using counting beads. Fluorochrome-labeled monoclonal antibodies were purchased from Biolegend (San Diego, CA), Thermo Fisher Scientific (Waltham, MA), or BD Pharmingen (San Jose, CA). FCM analysis was performed using an Aurora or LSRII (BD), and data was analyzed by FlowJo software (TreeStar, Ashland, OR). Details of antibodies used can be found in the **Supplementary Material**.

26-29 weeks post-transplantation, when HIS mice are expected to fully be reconstituted by human cells, we euthanized half of the animals in Experiment 1 and all the animals in Experiment 2, compared the cell populations within the spleen and thymus and also evaluated the size, structure, cellularity of the spleen and thymus tissues. A small piece of each lymphoid tissue, including spleen and thymus, was sent for histological studies to compare the structures of these tissues. Flow cytometry gating strategies for WBCs, thymocytes, and splenocytes is shown in **Figure S1**.

### Autoimmune disease

In all experiments, mice were scored for the development of autoimmunity weekly or biweekly until week 57 using a scoring system that evaluates body weight change, posture, hair coat and activity of the mice. Details of the scoring system are described in **Supplementary Material**. Animals with any signs of autoimmune disease (score greater than 2) were monitored daily and weights were recorded every other day. Animals with a total score of 6 or higher were monitored and weighed daily. Animals with a total score of 9 or higher or a score of 3 in any one category were euthanized, histology of lymphoid organs was performed and cell populations within spleen and thymus of all experiments were evaluated as described above for euthanized mice in Experiment 1.

### Statistical analysis

Statistical analyses were performed using GraphPad Prism 9.0 software (La Jolla, CA). Paired or unpaired Student’s t-tests (two-tailed) were used for analyses. A p value of <0.05 was considered to be statistically significant. The Bonferroni method was used to correct for multiple comparisons. Data are presented as median (horizontal line). Survival curves were generated using Kaplan-Meir plots with censoring.

## Results

### Human immune cell reconstitution and phenotype

**Figure 1:**
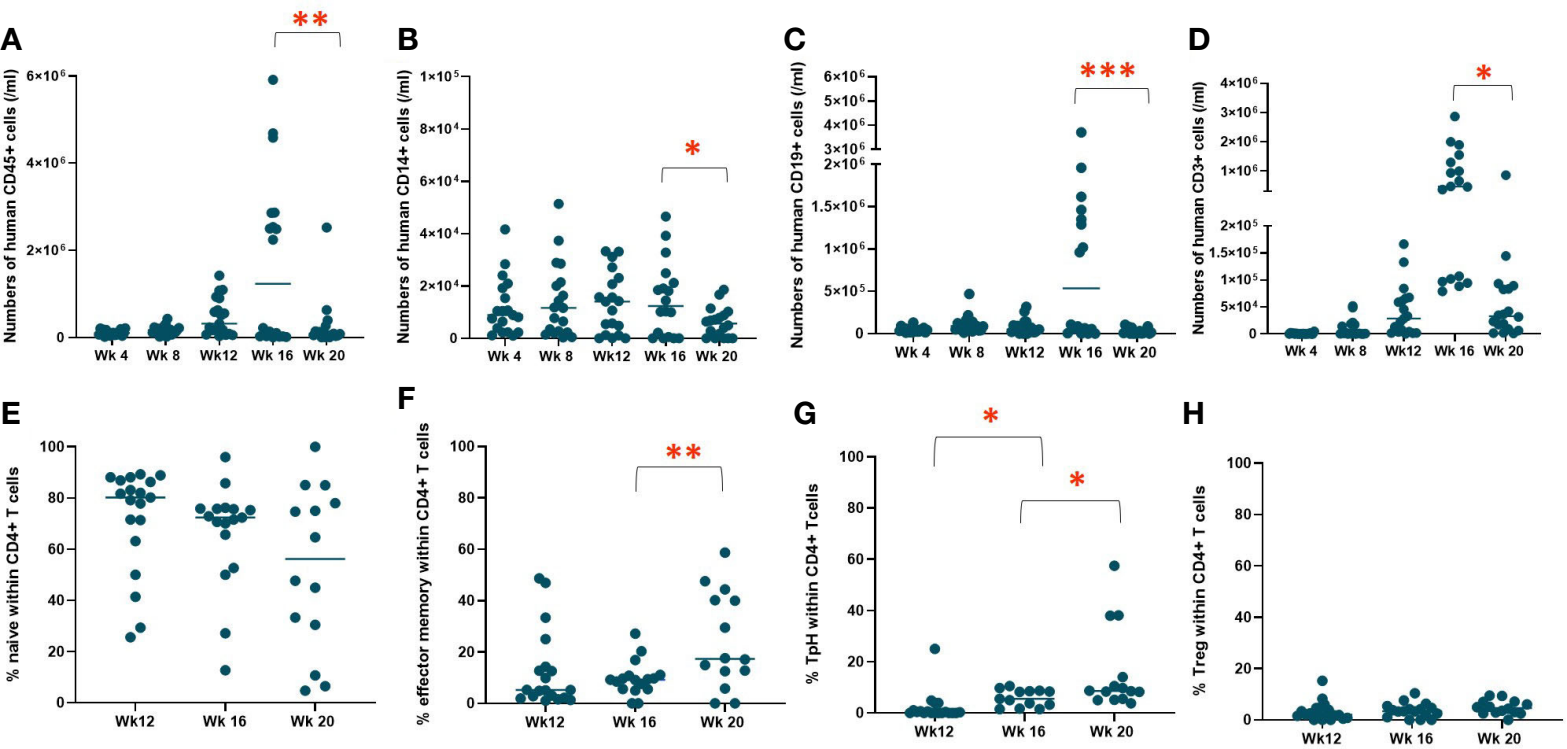
WBC reconstitution over time. HIS mice were generated with human neonatal thymus tissue and CB CD34^+^ cells. Following transplantation, human cell reconstitution was determined by analysis of peripheral blood by flow cytometry at different time points. Shown are numbers of **(A)** human CD45^+^ cells, **(B)** human CD14^+^ cells, **(C)** human CD19^+^ cells, and **(D)** human CD3^+^ cells per ml of peripheral blood and the median at each time point were calculated and plotted for group 1 (n=19). T cell phenotypes as percentages of CD4^+^ T cells, including **(E)** naïve, **(F)** effector memory, **(G)** Tph, and **(H)** Treg and the median at each time point were calculated and plotted for group 1 (n=19). *P< 0.05, **P<0.01, and ***P<0.001, by student’s t test for all comparisons.

To evaluate human immune cell reconstitution, we monitored the rate of repopulation and peripheral blood concentrations of T cells, B cells, and monocytes, including CD4^+^ and CD8^+^ T cells, naïve (CD45RA^+^CCR7^+^) and effector memory (CD45RA^-^CCR7^-^) CD4^+^ T cells, regulatory T cells (Tregs) (CD4^+^CD127^low^CD25^high^), and T peripheral helper (Tph) cells (CD4+CD45RA^-^CCR7^-^PD-1^+^CXCR5^-^) in the NeoHu mice in experiment 1 (n=19). **Figures 1A–D** demonstrate the reconstitution of human CD45^+^, CD14^+^, CD19^+^, and CD3^+^ cells, respectively, in the white blood cells (WBCs) over time. No mice had evidence of autoimmune disease at the time points shown in **Figure 1**, and one mouse died of an unknown cause without apparent autoimmunity during this time interval. All mice demonstrated reconstitution of hCD45^+^ cells and engraftment with human CD14^+^, CD19^+^, and CD3^+^ cells (**Figures 1A–D**), though variation in the level of reconstitution was observed. Between weeks 16 and 20, a statistically significant decline in overall human immune cell, monocyte, B cell, and T cell reconstitution was seen. Variation in human immune cell reconstitution was noted between different CB units, though group sizes were too small to assess for statistical significance (**Figure S2**). T cell phenotypes, including naïve, effector memory, Tph, and Tregs as percentages of CD4^+^ T cells were also analyzed over time (**Figures 1E–H**, respectively). The majority of CD4^+^ T cells initially demonstrated the naïve phenotype (**Figure 1E**). However, over time, there was a statistically significant increase in effector memory and Tph phenotypes, which we have previously observed in association with autoimmune disease development (6, 14).

**Figure 2:**
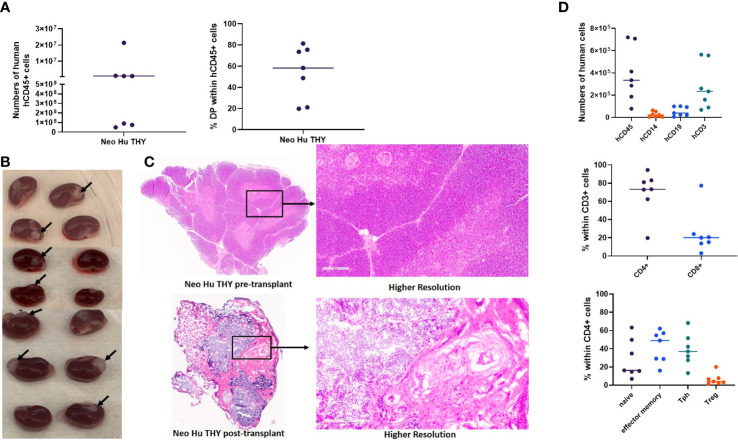
Thymic reconstitution. HIS mice were generated with human neonatal thymus tissue and CB CD34^+^ cells. 26-29 weeks post-transplantation, some disease-free HIS mice were euthanized and thymic grafts were isolated. Histology was performed and thymocytes were enumerated and analyzed by flow cytometry. **(A)** Total numbers of human CD45^+^ thymocytes (left) and percent CD4/CD8 double positive (DP) within human CD45^+^ thymocytes (right). **(B)** Gross view of neonatal human thymus grafts under the kidney capsule. Arrows point to the thymus grafts that were able to be harvested for histology analysis. **(C)** Representative H&E stain showing histology of one neonatal human thymus graft from one HIS mouse in Experiment#1. Histology of a piece of neonatal human thymus is included for comparison. **(D)** Numbers of cells (top) per ml of peripheral blood and percentages of naïve, effector memory, Tph, and Treg cells within CD4^+^ T cells in the spleen (bottom).

Twenty-six to twenty-nine weeks post-transplantation, when HIS mice are expected to be fully reconstituted by human cells, we euthanized 7 of the mice in Experiment 1 and compared the cell populations within the spleen and thymus. A small piece of spleen and thymus was used for histological studies to evaluate size, structure, and cellularity. All the mice in Experiment 1 at this time point were free of autoimmune disease. Thymic reconstitution (**Figure 2A**, left graph) and thymopoiesis as represented by the percentage of CD4/CD8 double positive (DP) thymocytes (**Figure 2A**, right graph) demonstrated variability. Variability was also seen in thymus graft size and thymic architecture, which did not appear normal in any graft, as clear cortical and medullary demarcation and Hassal’s corpuscles were not detected (**Figures 2B, C**). Two mice had no visible thymus graft tissue under one of the two kidney capsules (**Figure 2B**). Of note, only 7 of the 12 thymic grafts had sufficient remaining tissue to be sent for histology (**Figure 2C**) after tissue was taken for flow cytometric analysis. In the spleens (**Figure 2D** top graph), most human cells were T cells, with effector memory and Tph phenotypes most prominent within the CD4^+^ subset (**Figure 2D**, bottom panel).

### Origin of thymic and splenic T cells

**Figure 3:**
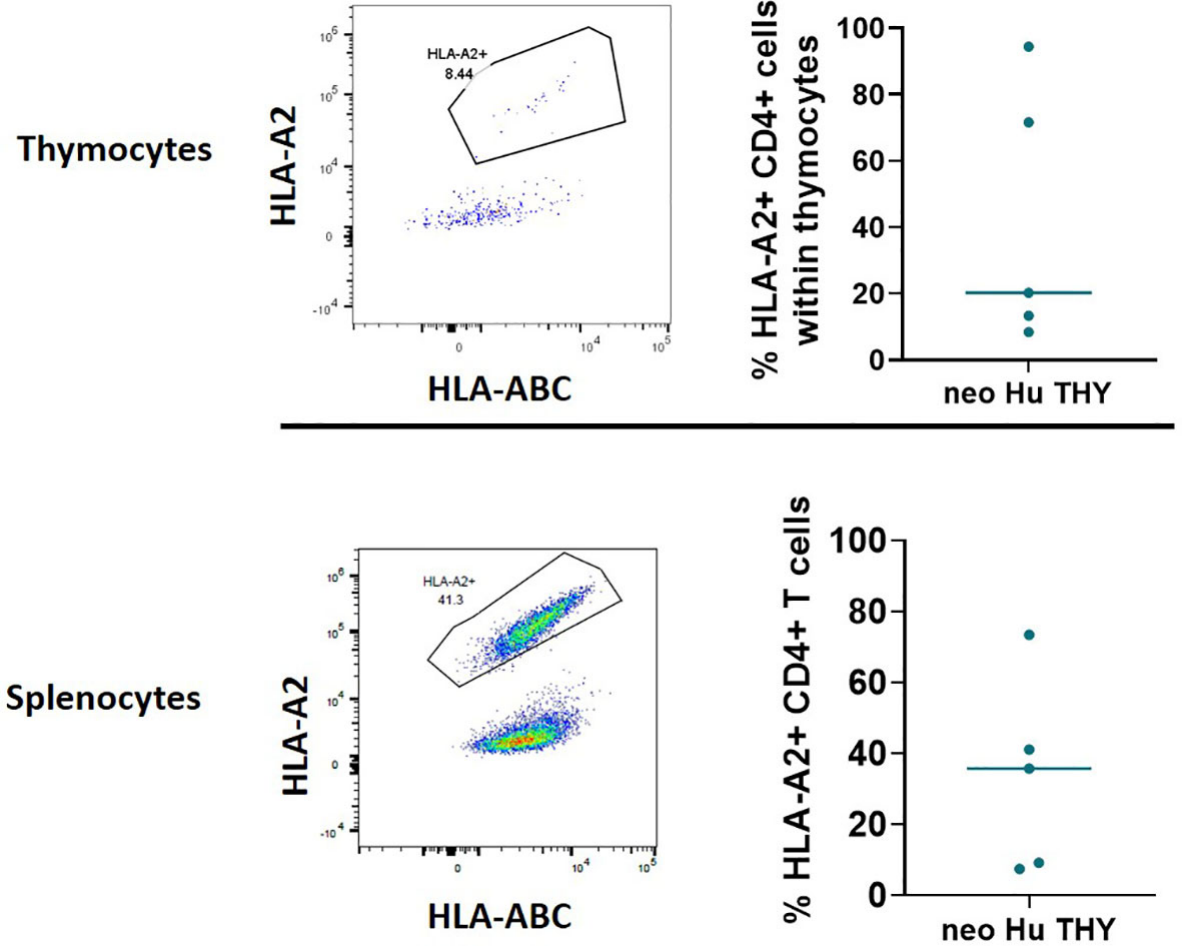
Origin of T cells within thymocytes (top) and splenocytes (bottom) using HLA markers in Experiment 1. HIS mice were generated with human neonatal thymus tissue and CB CD34^+^ cells. Thymic tissues were not treated with 2-DG. 26-29 weeks post-transplantation, some disease-free HIS mice were euthanized. Splenocytes and thymocytes from the thymic grafts were isolated and analyzed by flow cytometry. HLA-A2^-^ is the marker for CB derived T cells, and HLA-A2^+^ is the marker for thymus tissue derived T cells. Representative flow cytometry plots demonstrating HLA-A2- and HLA-A2^+^ CD4^+^ cells within thymocytes (top left) and splenocytes (bottom left). Top right and bottom right graphs demonstrate the percentages of HLA-A2^-^ or HLA-A2^+^ cells within CD4^+^ thymocytes and splenocytes, respectively, among all samples (n=5).

Previous studies in mice have demonstrated the presence of thymus-resident T cell progenitors that were able to self-renewed and support thymopoiesis without the input of T cell progenitors from hematopoietic stem cells (15, 16). Therefore, T cells in the HIS mice generated with neonatal thymus tissue may be derived from the neonatal thymus tissue itself or the CB-derived CD34^+^ cells. Using HLA markers, a portion of the same cohort of disease-free mice (n=5) from Experiment 1 that were euthanized at 26-29 weeks post-transplantation were evaluated for the origin (thymus vs CB) of T cells in the thymus and spleen (**Figure 3**). The HLA type of the CB was identified as HLA-A2^-^, while the HLA type of the neonatal human thymus graft was identified as HLA-A2^+^. T cells among CD4^+^ thymocytes and splenocytes were found to originate from two sources, both CB and thymus tissue (**Figure 3**). Similar results were also seen in Experiment 4 (described below). Similar to T cells in the spleen, both HLA-A2^+^ and HLA-A2^-^ thymocytes were found among CD4SP, CD8SP, and DP subsets in the thymic grafts (**Figure S3A**) and in peripheral blood and splenic CD8^+^ T cells (data not shown). No difference in the ratios of CD4/CD8 SP thymocytes derived from the thymus vs CB was seen (**Figure S3B**).

### Autoimmune disease development

**Figure 4:**
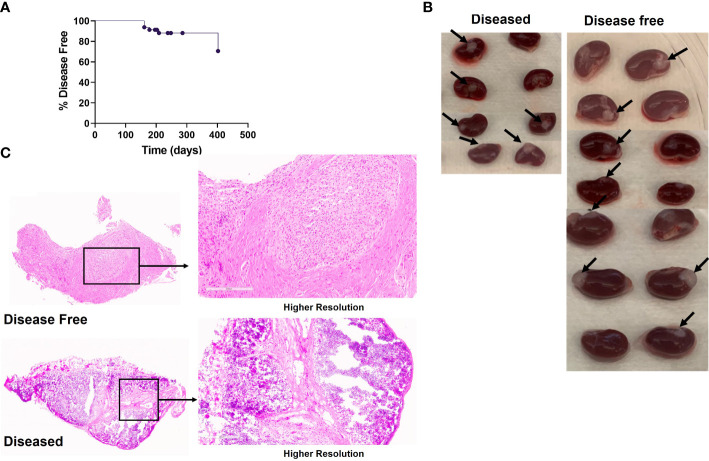
Autoimmune disease in the neonatal human thymus recipients. HIS mice were generated with human neonatal thymus tissue and CB CD34^+^ cells. HIS mice were monitored for autoimmune disease and autoimmune mice were euthanized when their clinical disease scores necessitated euthanasia. Disease-free mice were euthanized at the end of the study. **(A)** Onset of disease over time. Percent disease-free mice over time. Shown is the disease onset of these mice. **(B)** Gross histology of neonatal human thymus grafts under the kidney capsule, disease-free versus diseased, in Experiment#1. Arrows point to the thymus grafts that were able to be harvested from histology analysis. **(C)** Representative H&E stain showing histology of one neonatal human thymus graft from one diseased and disease-free HIS mouse in Experiment#1.

The remaining HIS mice in Experiment 1 (n=11), in which neonatal thymus tissue was not treated with 2-DG, were monitored up to 57 weeks to observe for the emergence of autoimmune disease. Onset of autoimmune disease was first noted at 23 weeks post-transplantation (**Figure 4A**), when a total of 4 mice showed evidence of the disease. Mice with autoimmune disease were euthanized and their tissues were analyzed when their autoimmune disease score was greater than 9. The remaining 7 disease-free mice were euthanized and processed at 57 weeks, when spleens and thymi were analyzed in the same way as for the cohort sacrificed earlier.

**Figure 5:**
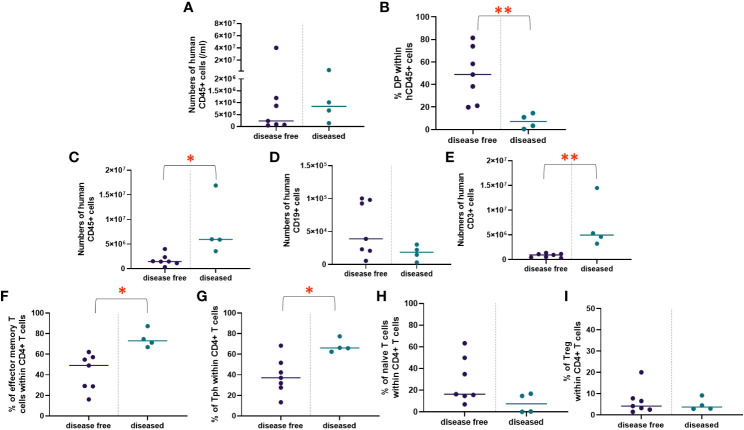
Thymopoiesis in disease free versus diseased mice in Experiment 1. HIS mice were generated with human neonatal thymus tissue and CB CD34^+^ cells. Thymic tissues were not treated with 2-DG. 11 mice were maintained for monitoring of autoimmune disease. Autoimmune mice were euthanized when their clinical disease scores necessitated euthanasia (n=4). Disease-free mice were euthanized at the end of the study (n=7). Human immune reconstitution among thymocytes of disease free versus diseased mice: **(A)** numbers of human CD45^+^ cells per ml of peripheral blood. **(B)** percentages of DP thymocytes within human CD45^+^ cells. Human immune reconstitution in splenocytes of diseased free mice versus diseased mice: numbers of human **(C)** CD45^+^, **(D)** CD19^+^ and **(E)** CD3^+^ cells. T cell phenotype among CD4^+^ splenocytes: **(F)** percentages of effector memory, **(G)** percentages of Tph, **(H)** percentages of naïve, and **(I)** percentages of Treg. Group 1 (n=11). *P< 0.05 and **P<0.01, by student’s t test for all comparisons.

Similar thymic graft size (**Figure 4B**) and similar lack of normal thymic architecture in the grafts (**Figure 4C**) were noted in the disease-free and diseased cohorts. Disease-free and diseased mice showed similar thymocyte counts and percentages of human CD45^+^ cells (**Figure 5A**). However, the diseased mice demonstrated a significant reduction in percentages of DP thymocytes compared to disease-free mice (**Figure 5B**). Furthermore, the numbers of human CD45^+^ cells in the spleen were significantly higher in the diseased mice compared to disease-free mice (**Figure 5C**). The numbers of B cells in the spleens of the two cohorts were similar, whereas the numbers of T cells were significantly higher in diseased mice, consistent with T cell lymphoproliferation (**Figures 5D, E**). Consistently, splenic T cells of diseased mice included significantly greater percentages of effector memory and Tph phenotypes (**Figures 5F, G**) compared to the disease-free mice. Percentages of naïve CD4 T cells and Tregs were similar and normal in both groups (**Figures 5H, I**).

### 2-DG effect on human immune cell reconstitution, T cell source, and autoimmune disease development

A decline in human immune cell reconstitution seen between Weeks 16 to 20, following early T cell appearance in peripheral blood between week 4 to 8 in Experiment 1, suggesting that pre-existing thymocytes released from neonatal thymus might have the potential to destroy allogeneic CB-derived cells and attack the recipient due to a lack tolerance. We then sought to eliminate thymocytes that developed in the original donor of the transplanted thymus grafts. We have previously demonstrated the ability of 2-DG to deplete pre-existing thymocytes from porcine fetal thymus tissue without impeding thymopoietic function (17). Therefore, the mice in Experiment 2 received thymic grafts treated with 2-DG, after which we evaluated human immune cell reconstitution and disease development over time.

**Figure 6:**
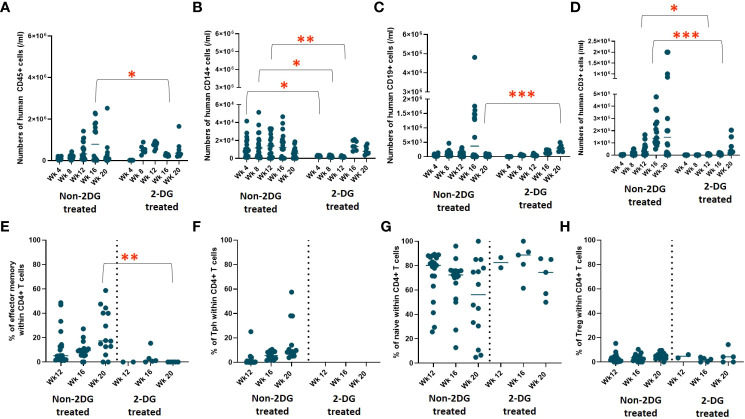
Effect of 2-DG on human immune cell reconstitution and T cell phenotype in WBCs over time. Two cohorts of HIS mice were generated with CB CD34^+^ cells and human neonatal thymus tissue that were either treated or not treated with 2-DG. Following transplantation, human cell reconstitution was determined by analysis of peripheral blood by flow cytometry at different time points. Shown are numbers of human **(A)** CD45^+^, **(B)** CD14^+^, **(C)** CD19^+^, and **(D)** CD3^+^ cells per ml of peripheral blood. T cell phenotype of non-2-DG versus 2-DG treated mice over time: **(E)** percentages of effector memory, **(F)** percentages of Tph, **(G)** percentages of naïve, **(H)** percentages of Treg within CD4^+^ T cells in non-2-DG and 2-DG treated mice. (group 1: n= 19; group 2: n = 7). *P< 0.05, **P<0.01, and ***P<0.001, by student’s t test for all comparisons.

**Figure 7:**
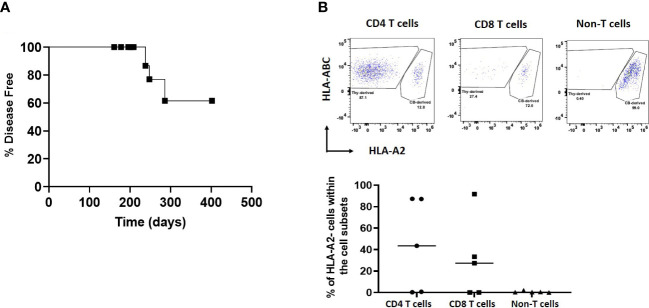
Autoimmune disease and origin of T cells in HIS mice receiving 2-DG-treated neonatal thymus tissue. HIS mice were constructed with 2-DG-treated neonatal thymus (Experiment #2 and Experiment #4). Autoimmune disease was monitored in Experiment #2 and origin of T cells in peripheral blood was determined by flow cytometry at week 20 in Experiment #4. **(A)** Onset of disease over time. HIS mice were monitored for autoimmune disease and autoimmune mice were euthanized when their clinical disease scores necessitated euthanasia. Disease-free mice were euthanized at the end of the study. Shown is the disease onset of these mice. **(B)** Source of T cells. HLA-A2^+^ indicates T cells derived from the CB, while HLA-A2^-^ indicates T cells derived from the thymus tissue. Data shown are from 5 mice with T cell reconstitution. One representative flow cytometric analysis data from one HIS mouse is shown in upper panel. Summarized data from 5 mice showing T cell reconstitution is shown in the lower panel.

When comparing 2-DG-treated to the non-2-DG-treated cohort, both groups had similar overall human immune cell reconstitution among WBCs, except at week 16 (**Figure 6A**). However, in contrast to the recipients of non-2-DG-treated grafts, recipients of 2-DG-treated grafts did not show a decline in human immune reconstitution at week 20 or early appearance of T cells between week 4 and 8 (**Figure 6A**). Monocyte (CD14^+^) reconstitution was significantly delayed (weeks 4-12) in the 2-DG-treated cohort (**Figure 6B**). B cell (CD19^+^) reconstitution was similar between the groups at all time points except for week 20, when it was significantly greater in recipients of 2-DG-treated grafts (**Figure 6C**). Furthermore, 2-DG treatment was associated with significantly reduced T cell reconstitution at weeks 12 and 16 compared to that in recipients of non-2-DG-treated grafts (**Figure 6D**). Circulating T cells in recipients of 2-DG-treated grafts showed significantly less effector memory phenotype compared to those in recipients of non-2-DG-treated grafts (**Figure 6E**). Moreover, circulating Tph cells were not detectable in recipients of 2-DG-treated grafts (**Figure 6F**). Percentages of naïve CD4 T cells and Tregs were similar in both groups (**Figures 6G, H**). Collectively, our data are consistent with a reduction in early T cell lymphoproliferation and the ability of human T cells to increase monocyte populations (7) in recipients of 2-DG-treated compared to untreated grafts. The first onset of autoimmune disease in the 2-DG-treated cohort was delayed (post-transplantation day 238) compared to that in Experiment 1 (post-transplantation day 161), though the ultimate disease incidence was similar between the two cohorts (**Figures 4A**, **7A**).

In Experiment 4, HLA-A2 was used as a marker to identify the source of T cells as either CB- derived or thymus tissue-derived among WBCs over time in recipients of 2-DG-treated grafts (n=10) (**Figure 7B**). HLA-A2^+^ T cells were derived from the CB, while HLA-A2^-^ T cells were derived from the thymus tissue. By the last time point evaluated (week 20), 5 mice did not show T cell reconstitution. Of the other 5 mice that showed T cell reconstitution, T cells derived from the thymus graft made up 0% to 87% and 91% of the CD4 and CD8 T cell compartment respectively. In contrast, non-T hematopoietic cells were exclusively derived from CB as expected (**Figure 7B**). These data show that treatment of neonatal thymus tissue was unable to completely remove thymus graft-derived human T cells.

### Variations in human immune cell reconstitution by neonatal human thymus tissue age

To evaluate the effect of the age of neonatal thymus tissue donors on human immune cell reconstitution, 2-DG-treated neonatal human thymus grafts from donors aged 2 weeks, 2 months, and 4 months were used in combination with FLC-derived CD34^+^ HSPCs to generate HIS mice in Experiment 3 (n=5 in each cohort). The rate of repopulation and concentrations of T cells as well as B cells and monocytes, including CD4^+^ and CD8^+^ T cells, naïve and memory CD4^+^ T cells in peripheral blood were evaluated as in Experiments 1 and 2.

**Figure 8:**
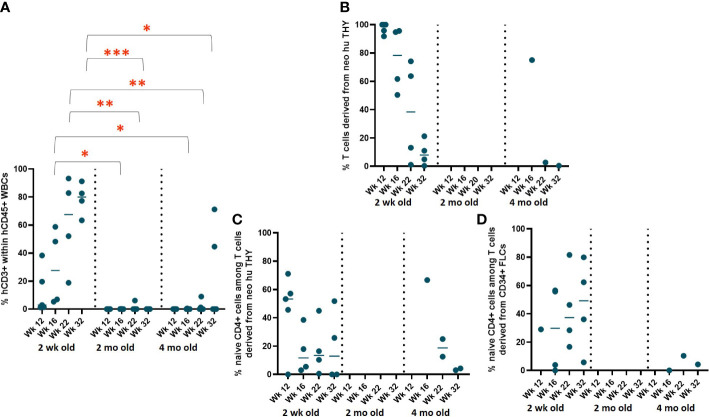
Variation in reconstitution by age of neonatal human thymus tissue. **(A)** T cell reconstitution over time. HIS mice were generated with FLCs CD34^+^ cells and human neonatal thymus tissues at different ages that were treated with 2-DG prior to implantation. Following transplantation, human cell reconstitution was determined by analysis of peripheral blood by flow cytometry at different time points. **(B)** Percent of T cells derived from the neonatal human thymus. **(C)** Percent naïve T cells among CD4^+^ T cells derived from neonatal human thymus and **(D)** CD34^+^ FLCs. *P< 0.05, **P<0.01, and ***P<0.001, by student’s t test for all comparisons.

While all surviving mice (4 of 4) receiving 2-week-old neonatal thymus showed T cell reconstitution, mice receiving 2-month-old neonatal thymus either did not show any T cell reconstitution (n=4) and only 2 of 5 recipients of 4-month-old thymic tissue showed circulating T cells (**Figure 8A**). Since we selected HLA-A2^+^ FLC as the source of CD34^+^ cells and HLA-A2^-^ thymic tissue in this experiment, we were able to distinguish the two potential sources of T cells. T cells in HIS mice receiving the 2-week-old neonatal thymus tissue demonstrated T cells from both the FLC CD34^+^ cells and the thymic tissue (**Figure 8B**), although the percentages of thymus-derived T cells tended to decline over time. In contrast, the few T cells in HIS mice receiving 4-month-old tissue were largely FLC CD34^+^ cell-derived. Furthermore, the percentages of naïve T cells among CD4^+^ T cells derived from the neonatal thymus tissues (2-week-old and 4-month-old) were similar over time (**Figure 8C**), while the percentage of naïve T cells among CD4^+^ T cells derived from FLCs was persistently greater in recipients of the younger neonatal human thymus tissue (2-week-old) compared to the older (4-month-old) (**Figure 8D**).

## Discussion

In this study we have attempted to reproduce and improve upon the neonatal thymus-based HIS mouse model described first by Brown et al. (8). The published paper did not rule out the native murine thymus as the site where human T cells developed, so we thymectomized the recipient mice to eliminate this confounding factor. Since human T cells do not develop in thymectomized NSG mice receiving human HSCs without a thymus graft (7), the studies here definitively demonstrate for the first time that human T cells developing in human neonatal thymus grafts can populate the periphery of HIS mice. Furthermore, we were able to demonstrate the presence of naïve T cells in the periphery, consistent with *de novo* thymopoiesis, whereas Brown et al. only detected effector/memory T cells. This difference likely reflects the greater amounts of neonatal thymus tissue implanted in our study compared to that used by Brown et al,. By using HLA markers to distinguish T cells originating in the neonatal thymus vs the CB CD34^+^ cells, we demonstrated that, despite exhaustive post-transplant treatment with T cell-depleting antibodies, cells carried in the neonatal thymus graft are a major source of early peripheral T cell population, and that these may destroy CB progenitor cells and may accelerate the development of GVHD/autoimmune disease. Treating neonatal thymus tissue with 2-DG to further deplete thymocytes reduced the contribution of thymus-derived T cells to the peripheral population and delayed the onset of autoimmune disease. Nevertheless, autoimmune disease developed in some mice in association with conversion of peripheral T cells to the effector/memory and T peripheral helper phenotypes.

We observed a sharp decline in human immune cell reconstitution at 20 weeks in mice with early appearance of peripheral T cells. Together with the demonstration that early-appearing T cells are thymus-derived, these results suggest that allogeneic CB HSCs may be rejected by non-tolerant T cells carried in the neonatal thymic tissue. Alternatively, the decline in human reconstitution may reflect eventual failure of the neonatal thymic grafts and the lack of peripheral human APC maintenance, which require peripheral T cells for maintenance in HIS mice (7). The mitigation of this loss of all 3 human cell lineages by using 2-DG treatment of the grafted thymic tissue favors the rejection explanation in the recipients of non-2-DG-treated grafts. Allogeneic CD34 cell rejection is likely accentuated with the use of neonatal thymic tissue in place of fetal tissue, as the more mature thymus presumably contains greater numbers of mature alloreactive T cells than second trimester fetal thymic tissue typically used in HIS mouse models. Indeed, we have successfully reconstituted mice with fetal thymic tissue and allogeneic adult BM CD34^+^ cells without 2-DG treatment (2, 13, 18), indicating that our standard measures for thymocyte depletion (freeze/thaw and physical dislodgement) and early-repopulating T cell depletion (anti-CD2) is sufficient to prevent rejection by the thymocytes contained in fetal thymic grafts. Additionally, because fetal thymic tissue has excellent growth potential, we are able to graft smaller amounts and obtain more robust T cell reconstitution than we have observed here using larger amounts of neonatal thymic tissue, which did not show significant growth or structural maintenance in our studies. Because we utilized larger amounts of neonatal thymus tissue (2 grafts, 2-4mm^3^), the total number of mature thymocytes grafted was likely greater than that in fetal tissue used in previous studies and greater than that in neonatal grafts used by Brown et al, who grafted 1mm x 1mm thymic fragments and did not see a loss of allogeneic human reconstitution at 20 weeks (8).

We previously demonstrated that fetal pig thymus has markedly greater growth potential and repopulating ability than neonatal pig thymus tissue in mouse models. While implanted fetal pig thymus grew markedly, supported excellent T cell reconstitution and persisted to 32 weeks post-transplantation (19), implanted neonatal pig thymus underwent initial growth, declined in size by week 7 post-transplantation and supported only limited T cell reconstitution (20). Increasing the amount of neonatal pig thymus tissue implanted partially corrected this limitation (21). Consistent with these previous findings, the overall level, quality and duration of human immune reconstitution in the NeoHu model is reduced compared to that using human fetal thymus tissue (1, 2, 6, 22), despite the use of increased amounts of neonatal thymus in our studies.

Thymocyte numbers in neonatal grafts were markedly lower than those in HIS mice prepared with fetal human thymus tissue and normal thymic structure was not observed with neonatal thymic grafts, in contrast to fetal grafts (6, 23). Similar to results using human fetal thymus tissue, T cells that initially reconstituted our NeoHu mice demonstrated a dominantly naïve phenotype. However, over time this phenotype was replaced by an increase in effector/memory and Tph phenotype and T cell expansion that we have associated with later autoimmune disease development in the fetal thymus recipients (6). Consistent with the early effector/memory/Tph conversion, autoimmune disease became apparent as early as (6)post-transplantation day 161 in animals receiving non-2-DG-treated grafts, prior to the time when it occurs in fetal thymus recipients (6). The rapidity of this phenotypic conversion in the NeoHu model compared to our historical results with fetal thymus and its mitigation by 2-DG treatment of the graft are consistent with antigen-driven expansion and/or lymphopenia-driven proliferation (LIP) of the low numbers of T cells migrating from the neonatal grafts in the NeoHu model. In other studies, we have found that LIP is a factor in the development of autoimmune disease in HIS mice (M. Khosravi and M. Sykes, manuscript in preparation). Given the lack of normal thymic architecture, it is also possible that tolerance to the thymus, the CB cells and/or the recipient mouse antigens may not develop normally in mice receiving neonatal thymus grafts. Although we did not investigate immune function in detail, Brown et al. reported robust immune function in their NeoHu model, including IgG in the plasma (8). We have observed human IgG in the plasma of mice with extensive T cell lymphoproliferation and the effector/Tph phenotype associated with development of autoimmunity (14) (Vecchione et al., manuscript in preparation). In aggregate, our observations suggest that the window for normal, non-autoimmune-driven immune responses is likely to be reduced in the NeoHu model compared to models using fetal human thymus tissue.

Significantly improved T cell reconstitution and a more naïve T cell phenotype were observed with two-week- old neonatal thymic tissue compared to 2-month and 4-month-old tissue. However, T cell reconstitution in mice receiving 2-month-old thymic tissues was poorer than that in mice receiving 4 months old tissues, suggesting that the reconstituting ability of neonatal thymus tissue becomes more unpredictable with increased age and younger tissues may be preferable to older neonatal thymus tissue in NeoHu mice models. However, the combined use of freeze/thawing, mechanical removal of thymocytes, 2-DG treatment and anti-CD2 antibody was not able to completely remove the 2-week-old neonatal thymus-derived T cells. Mixed chimerism in the T cell compartment significantly increases the complexity of the model.

While the NeoHu HIS mouse model is comparable to the human fetal thymus HIS mouse model in several important respects overall, it has yet to provide equivalently robust human thymopoiesis and naïve T cell reconstitution as that seen in mice receiving human fetal thymus tissue. Our studies suggest that further improvements in the NeoHu HIS mouse model would be needed in order to replace those generated with human fetal tissues for various applications in biomedical research.

## Data availability statement

The original contributions presented in the study are included in the article/**Supplementary Material**. Further inquiries can be directed to the corresponding author.

## Ethics statement

The animal study was reviewed and approved by Institutional Review Board and Animal Care and Use Committees at Columbia University.

## Author contributions

TT performed experiments, analyzed data, and wrote the manuscript. HL designed/performed experiments, analyzed data, and edited the manuscript. HW, W-IK, XD, ND, MG and AW performed experiments. MS directed the research and edited the manuscript. All authors contributed to the article and approved the submitted version.
