# Supplementary material for: Origin, phenotype and autoimmune potential of T cells in human immune system mice receiving neonatal human thymus tissue
Source: Front Immunol. 2023 May 12;14:1159341. doi: 10.3389/fimmu.2023.1159341 (PMC10213218; doi:10.3389/fimmu.2023.1159341)
Supplement: Supplementary file 1 [file DataSheet_1.pdf]

Supplementary material

Origin, phenotype and autoimmune potential of T cells in human immune system mice receiving neonatal human thymus tissue.

Tara Talaie, Hui Wang, Wan-I Kuo, Nichole Danzl, Mert R. Gulsen, Amber N. Wolabaugh, Xiaolan Ding, Megan Sykes, Hao Wei Li*

*** Correspondence:**

Hao Wei Li, Department of Medicine, Columbia Center for Translational Immunology, Columbia University Medical Center, 650 West 168th Street, Black Building 1511, New York, NY 10032, USA. Tel: 212.342.0237. Email: hl2591@cumc.columbia.edu

Animals, tissue preparation, and cord blood/FLCs processing

6 to 8 week-old female NOD-scid common cytokine gamma chain knockout (NOD.Cg-$Prkdc^{scid}$ $Il2rg^{tm1Wjl}$) (NSG) mice were obtained from the Jackson Laboratory (Bar Harbor, ME) and housed in a specific pathogen-free microisolator environment.

For Experiments 1 and 2, $CD34^+$ cells were isolated by using the human CD34 microbead kit (Miltenyi Biotech, Bergisch Gladbach, Germany).

All human tissue research was performed with approval from the Columbia University Institutional Review Board and performed in accordance with federal and state laws. Neonatal human thymus tissue was obtained with informed consent from newborn cardiac surgery patients during their care at Columbia University/New York Presbyterian Hospital. Immediately after surgical excision of the tissue, the thymi were placed in sterile bone marrow media (199 Media (Corning Inc, Corning, NY),1M HEPES (Life Technologies, Grand Island, NY), 1mg/ml DNAse (Sigma Aldrich), 50 mg/ml Gentamycin (Life Technologies, Grand Island, NY)), and surrounding non-thymic tissue was removed. Neonatal human thymus fragments were cryopreserved in 10% dimethyl sulfoxide and 90% human AB serum (Atlanta Biologicals). Cryopreserved human neonatal thymus fragments measuring about 1-4 mm^3 were thawed and transplanted under one or bilateral kidney capsules of these mice, as described[6]. In addition to freeze/thawing the thymus tissues to remove thymocytes as described[6], we also mechanically removed residual cells by repeated pipetting up and down before transplantation.

In Experiment 3, prior to thymus transplantation, the human neonatal thymus tissue was also incubated with 2-DG (1 thymus piece/well in 96 well plate, 200µl of 100 mM 2-DG (Sigma

Aldrich) in AIM V media with 10% human serum/well (Life Technologies, Grand Island, NY)) for 16-hours prior to transplantation for further removal of native thymocytes.

Generation of HIS mice

The Institute of Comparative Medicine (ICM) at Columbia University approved all experiments. To further deplete passenger thymocytes that might migrate to the periphery and limit allogeneic HSC engraftment, an anti-primate CD2 antibody (LoCD2b) was injected in mice in all three experiments in three doses (400µg/mouse, intraperitoneal) as we have described[6]. All mice were treated with Buprenorphine for post-operative pain control.

We generated 4 experiments of HIS mice using neonatal human thymus, either treated or not treated with 2-DG, plus $CD34^+$ HSCs derived from either CB cells or FLCs as detailed in Table 1. For analysis of human reconstitution, mice were bled at regular intervals for FCM analysis of human T cells, B cells and monocytes and their naïve/memory phenotype.

Human immune cell reconstitution assessment

Peripheral mouse blood

The WBCs of the mice was sampled at weeks 4, 8, 12, 16, and 20 via tail vein bleed into heparin-coated capillary tubes (Thermo Fisher Scientific, Waltham, MA). The red blood cells were lysed with ACK red blood cell lysis buffer (Thermo Fisher Scientific, Waltham, MA) followed by wash with FACS buffer (10 mM HEPES-buffered Hank's balanced salt solution (Thermo Fischer Scientific, Waltham, MA), 2% fetal bovine serum (Thermo Fisher Scientific, Waltham, MA), 0.1% Sodium Azide (Sigma Aldrich), and spun down at 1680 RPM x 5 minutes).

Thymus and Spleen

Thymus and spleen grafts were processed from euthanized HIS mice through a 70µm cell strainer (Corning Inc, Corning, NY) with the blunt end of a 3ml syringe (BD Biosciences, San Jose, CA) into a petri dish containing sterile bone marrow media. Splenocytes underwent additional processing with ACK buffer for lysis of RBCs. Isolated cells from both thymi and spleens were collected and washed with FACS buffer. Live cell numbers were determined via hemocytometer counting after trypan blue staining.

Flow cytometric (FCM) analysis was performed to determine percentages and absolute counts of each population as previously described[12]. Absolute numbers of each population were calculated using counting beads. A combination of the following antibodies were used in flow cytometry to detect specific T cell markers: anti-human (αh) CD45RA (clone HIT100, 304114), αhCCR7 (clone G043H7, 353204), αhPD-1 (clone EH12.2H7, 329914), anti-mouse CD45 (clone 30-F11, 557659), αhCXCR5 (clone RF8B2, 562747), αhCD8 (clone RPA-T8, 563822), αhCD25 (clone 2A3, 563159), αhCD3 (clone OKT3, 317330), αhCD45 (clone HI30, Q10156), αhCXCR3 (clone 1C6, 566532), αhCD4 (clone RPA-T4, 560768), αhCD127 (clone A019D5, 351308), αhHLA-ABC (clone G46-2.6, 555555), αhHLA-A2 (clone BB7.2, 343304), αhHLA-BW6 (clone REA143, 130-123-264), and Zombie NIR Fixable Viability Dye (423105). A combination of the following antibodies were used in flow cytometry to detect specific B cell markers: αhCD45 (clone HI30, 560777), anti-mouse CD45 (clone 30-F11, 560501), αhIgM (clone MHM-88, 314544), αhIgD (clone IA6-2, 348232), αhCD20 (clone 2H7, 302310), αhCD138 (clone MI15,

356513), αhCD14 (clone M5E2, 301820), αhCD27 (clone O323, 302834), αhCD24 (clone ML5, 311104), αhCD38 (clone HIT2, 303516), αhCD3 (clone UCHT1, 300408), αhCD19 (clone HIB19, 302254), and Propidium Iodide (13-6990-T500).

Fluorochrome-labeled monoclonal antibodies were purchased from Biolegend (San Diego, CA), Thermo Fisher Scientific (Waltham, MA), BD Pharmingen (San Jose, CA), Miltenyi Biotech (Bergisch Gladbach, Germany), or Tonbo Biosciences (San Diego, CA). FCM analysis was performed using a Aurora or LSRII (BD), and data was analyzed by FlowJo software (TreeStar, Ashland, OR). Dead cells and doublets were gated out in all experiments. The gating strategy for T cell phenotypes within PBMCs, splenocytes, and thymocytes is demonstrated in Figure S1.

Autoimmune disease monitoring

In all experiments, mice were scored for the development of autoimmunity once per week until week 57 using the scoring system below:

Weight loss (%): $<10\%$, 0; $<10-15\%$, 1; $<15-20\%$, 2; $>20\%$, 3

Posture: Normal, 0; Mildly hunched at rest, 1; Moderately hunched, able to ambulate normally, 2; Severe hunching, impairs movement and gait, 3

Hair coat: Normal, 0; Mild ruffling or alopecia, 1; Moderate ruffling or alopecia, 2; Severe ruffling or alopecia, 3

Activity: Normal, 0; Mild to moderately decreased, 1; Active only to eat, drink or when stimulated, 2; difficulty rising, unable to move when stimulated, 3

Histology

A small piece of each lymphoid tissue, including spleen and thymus, was sent for histological studies to compare the structures of these tissues. Tissues were collected, fixed in 10% neutral buffered formalin and submitted for histological analysis by the Columbia Center for Translational Immunology Histology Core and processed into paraffin blocks, cut into 5 µm sections, and stained with routine Hematoxylin & Eosin (H&E).

Supplementary Figures

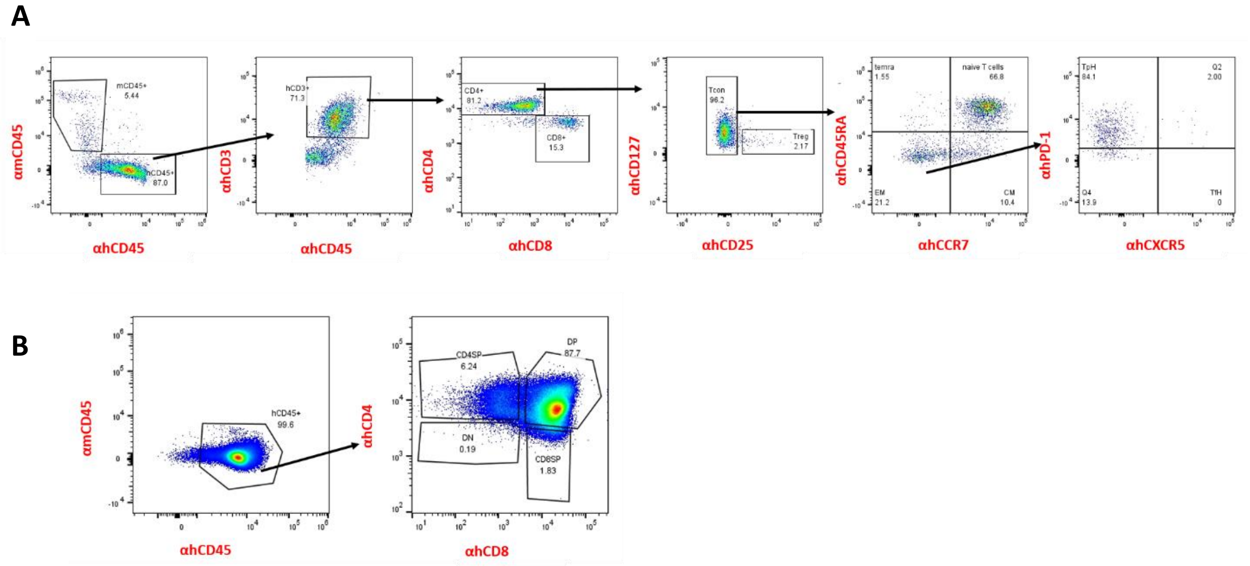

Figure S1. Flow cytometry gating strategies for T cell phenotypes within A) peripheral white blood cells (WBCs) and splenocytes, B) thymocytes.

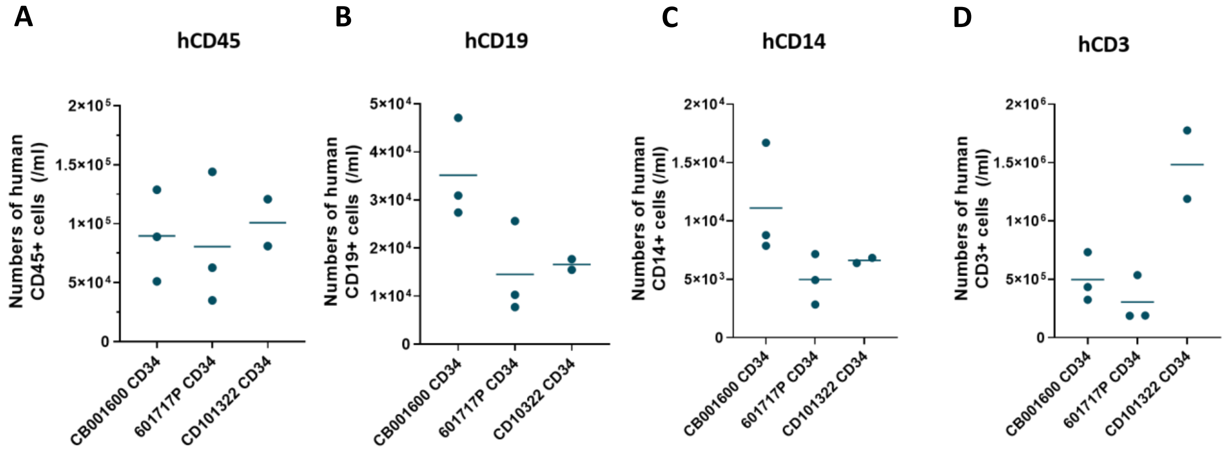

Figure S2. Variation by cord blood in reconstitution. HIS mice were generated with human neonatal thymus tissue and CB CD34$^+$ cells (Experiment 1). CD34$^+$ cells from several units of CB were used for the generation of HIS mice. Following transplantation, human cell reconstitution was determined by analysis of peripheral blood by flow cytometry at different time points. Human cell reconstitution by different units of CB is shown: a) human CD45$^+$, b) human CD19$^+$, c) human CD14$^+$, and d) human CD3$^+$ cells per ml of peripheral blood at week 20 post-transplantation.

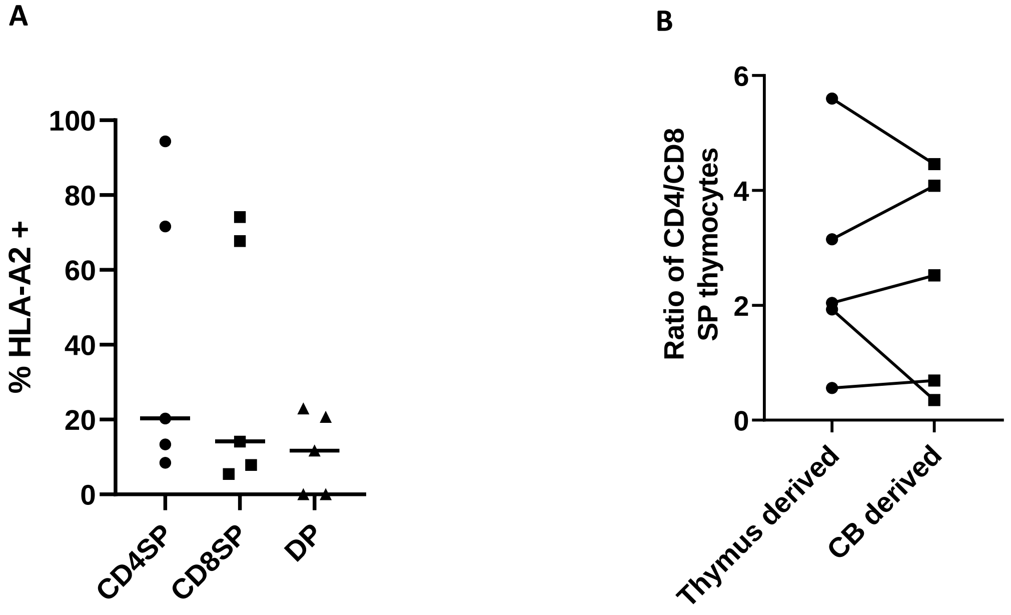

Figure S3. Thymic reconstitution by neonatal thymus and CB derived progenitors. HIS mice were generated with human neonatal thymus tissue and CB $CD34^{+}$ cells. Thymic tissues were not treated with 2-DG. a.) Thymocytes were analyzed for percentage of $HLA\text{-}A2^{+}$ (thymus derived) CD4SP, CD8SP, and DP thymocytes (Experiment 1, disease free: n=5). Median in each group is plotted (horizontal line). No significant differences were found among the groups. b.) Ratios of CD4/CD8 SP thymocytes derived from thymus ($HLA\text{-}A2^{+}$) and CB ($HLA\text{-}A2^{-}$) were shown. No significant differences were found between the two groups by paired T test.
